# Modeling Human Cardiac Arrhythmias: Insights from Zebrafish

**DOI:** 10.3390/jcdd9010013

**Published:** 2022-01-05

**Authors:** Sébastien Gauvrit, Jaclyn Bossaer, Joyce Lee, Michelle M. Collins

**Affiliations:** Department of Anatomy, Physiology, and Pharmacology, College of Medicine, University of Saskatchewan, Saskatoon, SK S7N 5E5, Canada; gauvritsebastien@gmail.com (S.G.); jaclyn.bossaer@usask.ca (J.B.); jol299@mail.usask.ca (J.L.)

**Keywords:** cardiac arrhythmia, heart development, zebrafish, genetic models, atrial fibrillation, inherited arrhythmia, cardiomyopathy, in vivo screening, imaging, cardiac rhythm phenotyping

## Abstract

Cardiac arrhythmia, or irregular heart rhythm, is associated with morbidity and mortality and is described as one of the most important future public health challenges. Therefore, developing new models of cardiac arrhythmia is critical for understanding disease mechanisms, determining genetic underpinnings, and developing new therapeutic strategies. In the last few decades, the zebrafish has emerged as an attractive model to reproduce in vivo human cardiac pathologies, including arrhythmias. Here, we highlight the contribution of zebrafish to the field and discuss the available cardiac arrhythmia models. Further, we outline techniques to assess potential heart rhythm defects in larval and adult zebrafish. As genetic tools in zebrafish continue to bloom, this model will be crucial for functional genomics studies and to develop personalized anti-arrhythmic therapies.

## 1. Introduction

The healthy human heart beats with a coordinated rhythm. Abnormal heart rhythm, or arrhythmia, refers to conditions in which heart rate or rhythmicity are altered or chaotic. The main inherited cardiac arrhythmias are long QT syndrome (LQTS), short QT syndrome (SQTS), catecholaminergic polymorphic ventricular tachycardia (CPVT), and Brugada syndrome (BrS). These diseases often result from mutations in genes encoding ion channels, leading to altered ionic currents that influence the cardiac action potential [1,2]. The most common cardiac arrhythmia is atrial fibrillation (AF), which is frequently associated with aging, inflammation, or following surgery [3,4,5]. A portion of AF cases arise in the absence of predisposing factors, often with a younger age of onset and with significant heritability [6]. Arrhythmias also occur in conjunction with inherited cardiomyopathies, including hypertrophic cardiomyopathy (HCM), dilated cardiomyopathy (DCM), arrhythmogenic cardiomyopathy (ACM), and left ventricular non-compaction cardiomyopathy (LVNC). These cardiomyopathies are frequently associated with mutations in genes encoding sarcomeric, desmosomal, or cytoskeletal proteins.

While often considered purely electrical diseases, primary cardiac arrhythmias have variable aetiologies. Genome-wide association studies (GWAS) and whole exome/genome sequencing techniques have implicated diverse pathways in the pathogenesis of cardiac arrhythmia, including developmental [7] and structural genes [8,9,10]. Many of the loci identified in GWAS are found in non-coding regions, suggesting that these variants alter gene expression which confers disease susceptibility [11,12]. Finding the genes that are affected by variants, and understanding their biological relevance in disease, is critical. The use of in vitro studies [13], animal models, and in silico modeling approaches [14] to investigate pathways implicated in cardiac arrhythmia has started to uncover some of the pathophysiological mechanisms of cardiac arrhythmias.

The zebrafish has emerged as an exceptionally powerful model to study cardiac development and disease. From a practical perspective, zebrafish are optically transparent and develop externally, enabling observation during development. They have high fecundity such that large numbers of embryos are easily acquired. Manipulating the zebrafish genome is relatively straightforward, and numerous reporter lines allow for visualization of cellular and organ-level morphology and physiology. Notably, the small size of zebrafish enables them to survive without a functional cardiovascular system early in development, as their oxygen and nutritional needs can be met by diffusion into the embryo. This advantage permits analyses of mutants with little to no cardiac function [15], whereas orthologous mutants in other vertebrate models would not survive long enough to be observed.

Here, we review the major milestones in zebrafish cardiac development, examine the suitability of using zebrafish to study the conduction system, review models of cardiac arrhythmia that have shed insights into the basis of human cardiac arrhythmias, and revisit methodologies to assess cardiac function in zebrafish.

## 2. Heart Development in Zebrafish

**Figure 1:**
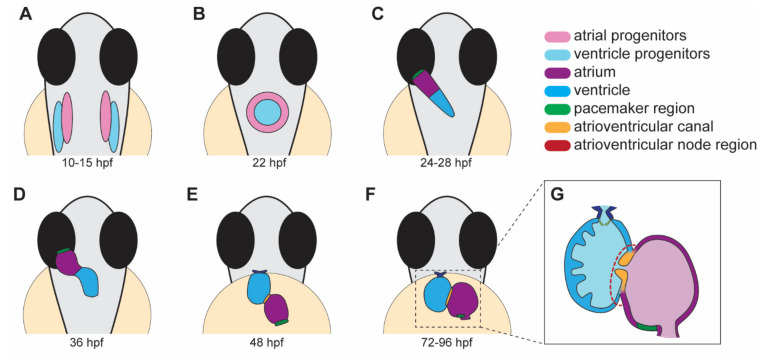
Stages of heart development in zebrafish from 10 to 96 h post-fertilization (hpf). (**A**) Atrial and ventricular cardiac progenitors are located in the anterior lateral plate mesoderm by ~15 hpf. (**B**) The cardiac disc is visible by 22 hpf as cardiac progenitors surround endocardial cells at the midline. (**C**) At 24 hpf, the linear heart tube forms and jogs to the left in preparation for heart looping. (**D**) At 36 hpf, the heart tube undergoes rightward looping. The AV canal (orange) begins to develop between the cardiac chambers. (**E**) At 48 hpf, the cardiac chambers begin to balloon and expand outwards. The bulbus arteriosus (dark blue) and AV canal (orange) continue to develop and mature. (**F**) From 72 to 96 hpf, the cardiac chambers expand and align beside each other. (**G**) A cross-section of a 96 hpf heart showing the trabeculae, the finger-like muscular projections on the inner wall of the ventricle, and endocardial leaflets (orange) of the AV canal. (**A**–**D**) dorsal views; (**E**–**G**) ventral views.

Heart development in zebrafish involves several distinct steps. This section will briefly outline the major processes that occur during each stage of heart development (Figure 1).

### 2.1. Migration and Differentiation of Cardiac Progenitors for the Formation of the Cardiac Disc

Heart development begins with the specification of myocardial progenitors in the lateral marginal zone at the 40% epiboly stage (~5 h post-fertilization, hpf). Chamber progenitor cells are derived from distinct progenitor pools, with ventricular progenitor cells located more marginally and dorsally compared to atrial myocardial progenitors [16,17,18]. By early somitogenesis (10–15 hpf, Figure 1A), bilateral pools of atrial and ventricular cardiac progenitors are positioned in the anterior lateral plate mesoderm [18,19]. These bilateral pools migrate towards the midline at the 26-somite stage (~22 hpf, Figure 1B), where they fuse to form the cardiac disc with ventricular precursors surrounded by atrial precursors [17,18,20]. Differentiation of cardiac progenitor cells can be identified by the expression of chamber-specific genes such as *ventricular myosin heavy chain (vmhc)* in the ventricular progenitors at around the 13-somite stage (~15 hpf) [17] and *atrial myosin heavy chain (amhc)* in the atrial progenitors by the 19-somite stage (~16 hpf) [21].

A network of transcription factors, including GATAs, NKX2.5, and HAND2, controls the differentiation of cardiac progenitors. The role of the Gata transcription factors was highlighted by the discovery of the *faust* zebrafish mutant carrying a mutation in *gata5* [22], which have reduced numbers of cardiac progenitor cells. Progenitors are completely lost in *gata5*/*gata6* morphants, suggesting that Gata factors are complementary for this process [23]. *NKX2-5* is required for cardiac development in multiple organisms [24] including human [25]. In zebrafish, two homologs are present, *nkx2.5* and *nkx2.7*. *nkx2.5* mutants show increased atrial and decreased ventricular cell numbers while *nkx2.5*/*nkx2.7* double mutants display a stronger phenotype demonstrating the synergy between both factors to maintain ventricular identity [26]. Hand2, a basic helix-loop-helix transcription factor regulates the number of myocardial progenitors. Zebrafish *hand2* mutants present a substantial reduction in the myocardial cell number [27]. Additional signaling pathways regulate the number of cardiac progenitors, including Bmp [28], Nodal [16,29], Wnt [30], fibroblast growth factors [31,32], and retinoic acid [33,34], highlighting that multiple pathway interactions are needed to provide the necessary number of cardiac progenitors (for more details of the molecular mechanisms, see reviews [35,36,37]).

### 2.2. Heart Jogging and Looping

The cardiac disc elongates to form a linear tube. At 24 hpf, the linear heart tube extends and jogs in an anterior and leftward direction (Figure 1C) [38,39]. This process is necessary for the morphogenesis and robustness of the later process of cardiac looping. If the linear heart tube jogs incorrectly to the right or remains at the midline, the probability of the heart correctly looping afterwards decreases [39]. Heart looping occurs around 36 hpf and corresponds to the rightward bending and twisting of the linear heart tube to create an S-shaped tube with the ventricle positioned to the right of the atrium (Figure 1D). Both intrinsic and extrinsic factors regulate heart looping. A heart cultured in vitro will still undergo heart looping, demonstrating that intrinsic factors play a role in heart looping [40,41,42,43,44,45]. Blood flow is an example of an extrinsic factor that induces elongation of the ventricular cardiomyocytes in the outer curvature [46]. These cellular shape changes are important for the bending the linear heart tube into an S-shaped tube.

### 2.3. Chamber Ballooning

Correct cardiac function requires chambers to emerge from the linear heart tube. At 48 hpf, the atrial and ventricular chambers bulge and expand during a process known as chamber ballooning (Figure 1E). This process is driven by changes in cell shapes within the inner and outer curvatures of the developing chambers, as well as cytoskeletal rearrangements [46,47]. Regionalized cellular elongation in the outer curvatures and compaction in the inner curvatures give the chambers balloon-like shapes. Biomechanical forces, including contractility and blood flow, also contribute to chamber ballooning. Blood flow induces cuboidal cells in the linear heart tube to elongate and expand to form the outer curvatures [46]. Additionally, extracellular matrix (ECM) proteins are crucial during chamber ballooning. Loss of hyaluronan and proteoglycan link protein 1a (Hapln1a), which cross-links hyaluronan with proteoglycans, reduces atrial size and chamber ballooning [48]. Interestingly, the expression of *hapln1a* is highest in the future atrium and on the left side of the linear heart tube, which is the same region with an expansion of the ECM between the myocardium and endocardium layers before heart looping and chamber ballooning occur. Recent data has shown that crosstalk between the endocardium and myocardium contributes to cardiac morphogenesis, as the expansion of the atrial chamber triggers the proliferation of the endocardium [49]. This mechanism is mediated by biomechanical signaling that is triggered by increased tensile forces within endocardial cell junctions.

### 2.4. Atrioventricular Valve Formation

Cardiac valves emerge from the atrioventricular canal (AVC), located between the atrium and ventricle (Figure 1F,G). During development, the AVC endocardium is remodeled into mature valves that block retrograde blood flow [50,51,52]. Endocardial cells first invade the ECM between endocardial and myocardial layers in the AV region and differentiate to give rise to the valve interstitial cells. Subsequent remodeling and leaflet elongation leads to mature valves. Communication between the endocardium and myocardium in the AVC is important for proper valve development. Early specification of valve endocardial cells is mediated by Bmp, Notch, NFAT, and ErbB signaling [52,53,54]. The ECM plays a critical role in promoting valve formation, acting as an active substrate for cell behaviour via the integrin-mediate focal adhesion complex [50]. While it has been recognized for some time that mechanical forces are required for valve formation, new findings have shed light on how forces are interpreted by the cell. These elegant studies show that regionalized shear stress forces occurring specifically in the AVC region are transformed into bioelectric cues via an ATP-mediated Ca^2+^ flux and Nfatc1 activation [55].

### 2.5. Trabeculation

Trabeculae are transverse muscular ridges in the inner wall of the ventricle that increase cardiac output and oxygen uptake of the myocardium without increasing heart size [56,57]. At the end of heart looping, a subset of cardiomyocytes delaminates from the outer curvature of the ventricular compact layer towards the lumen and form trabeculae (Figure 1F,G) [57,58,59]. Neuregulin/Erbb2 and Notch pathways cooperate to promote trabecular emergence, as zebrafish lacking *nrg2a* [60] or *erbb2* [57] fail to form trabeculae. Mechanistically, the endocardium secretes the ligand Neuregulin which activates its receptor, Erbb2, expressed by the myocardium. Erbb2 signaling also activates glycolysis to rapidly fuel ATP to the cardiomyocytes undergoing cellular shape changes to form the trabecular layer [61]. Loss of Notch signaling also impairs trabeculation [62,63]. Notch signaling is activated in compact layer cardiomyocytes adjacent to the cardiomyocytes that will delaminate to seed the trabecular layer. Blocking Notch signaling increases the number of delaminating cardiomyocytes, suggesting that Notch antagonizes trabecular emergence by lateral inhibition [62]. As described below, ventricular trabeculae are important structures for the fast conduction system in zebrafish.

### 2.6. Cardiac Conduction System Development

The development of the zebrafish cardiac conduction system (CCS) occurs in four stages. A linear conduction path is evident by 24 hpf, which travels from the sinus venosus to the ventricular outflow tract [64]. Optical mapping studies revealed that action potentials propagate slowly across the linear heart tube [65]. Unidirectional activation at this early stage suggests that sinoatrial node (SAN) pacemaker activity is already present [64,65,66]. From 36 to 48 hpf, impulse propagation substantially increases, and atrioventricular (AV) conduction delay develops. Optogenetic studies identified that the pacemaker region is confined to the sinoatrial ring [66], and pacemaker cells within this region express the LIM-homeodomain transcription factor Isl1 [67]. In the third stage, from 72 to 96 hpf, the ventricle develops an immature fast conduction network as ventricular trabeculation emerges [64,68]. The pacemaker region becomes further refined at this stage to the dorsal right quadrant (Figure 1F,G), and AV blocks can be induced through optogenetic manipulation of the AVC region [66]. Moreover, electrical gradient heterogeneity emerges between the inner and outer curvature of the myocardium [65]. Maturation of the fast conduction network is evident by 2–3 weeks post-fertilization when the ventricular apex has formed [64].

**Figure 2:**
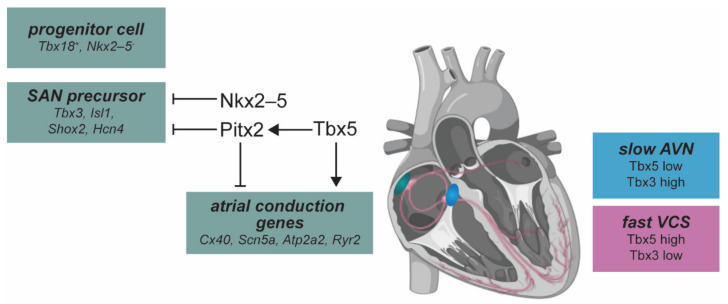
Overview of molecular pathways in the mammalian cardiac conduction system. The sinoatrial node (SAN, green) develops from *Tbx18^+^, Nkx2-5^low^* progenitor cells. *Nkx2-5* represses expression of *Tbx3* and *Isl1* to establish the boundary between the SAN and atrial cardiomyocytes. SAN precursor cell differentiation is marked by expression of *Tbx3, Isl1, Shox2,* and *Hcn4.* Pitx2 represses SAN development on the left side of the sinus venosus by repressing this transcriptional network. The Tbx5 (activator) and Pitx2 (repressor) regulatory loop regulates atrial conduction genes. Relative Tbx3/Tbx5 dosage determines specification of the conduction system. The atrioventricular node (AVN, blue) acts as a secondary pacemaker, and is characterized by slow conduction that is patterned by low levels of Tbx5 and high levels of Tbx3. A fast conduction state in the ventricular conduction system (VCS, pink) is specified by higher levels of Tbx5 and low Tbx3.

The CCS is derived from cardiomyocyte progenitors which give rise to two classes of conduction tissue: slow conducting tissue with longer refractory periods and a fast conducting tissues with rapid impulse propagation [69]. The transcriptional network that drives CCS development is highly conserved across evolution and has been elucidated from mouse models (for a detailed review, please see [70]) (Figure 2). The SAN develops from *Tbx18^+^*, *Nkx2-5^low^* progenitor cells. Pitx2, a key player in early cardiac patterning, inhibits the SAN-specific genetic network in the left atrium, which includes the homeobox transcription factor Shox2, LIM homeodomain factor Islet1, and Tbx3 [71,72,73]. The SAN forms from *Nkx2-5*^−^ precursors, and *Nkx2-5* supresses *Tbx3* and *Hcn4* expression to establish and maintain the boundary between the SAN and atrial myocardium [74]. Shox2 promotes SAN fate by repressing *Nkx2*-5 expression [75] and maintains expression of *Islet-1* (*Isl1*) specifically in the SAN [76,77,78]. Tbx5 directly regulates the expression of *Pitx2.* Tbx5 and Pitx2 antagonistically regulate several ion channel genes that are important for cardiac ion currents, including *Scn5a*, *Cx43* (*Gja1*), and *Atp2a2* [79]. The ventricular fast conduction system is patterned by a balance between the transcriptional activator Tbx5, and the repressor Tbx3. Tbx3 expression is enriched in the slow-conducting AVN, whereas Tbx5 is enriched in the fast ventricular conduction system [80]. Endocardial signaling is required for the development of AV conduction tissue, where Notch and Neuregulin pathways contribute to the formation and induction of slower conducting tissue [69]. Semaphorin signaling acts at various points in cardiovascular development [81] including during epithelial–mesenchymal transition in the outflow tract and AVC regions, cardiac innervation, and myocardial wall morphogenesis [82].

## 3. The Zebrafish Conduction System

The CCS is composed of pacemaker cells in the SAN, the atrioventricular node (AVN), and the fast ventricular conduction system. Although the zebrafish heart is formed by only a single atrium and single ventricle, similarities between its CCS with the human CCS supports the use of zebrafish as a model to study CCS development and function/dysfunction. Pacemaker cells have been identified in the sinus venosus and atrium junction [67], and slow-conducting AVC cardiomyocytes in the AVN region have been mapped in zebrafish [66]. While the mammalian His-Purkinje system is absent in zebrafish, the ventricular trabecular myocardium has been postulated to serve as a functional equivalent [68].

The molecular profiles of conducting tissues are highly conserved in zebrafish. Tomo-seq, a technique to spatially resolve genome-wide transcriptomics data, was used to profile the 2 days post-fertilization (dpf) zebrafish heart. These data identified a sub-compartment that highly expresses pacemaker development genes, including *isl1* and *shox2* [83]. Recent transcriptome profiling of the sinoatrial ring [84] and AVC cells [85] from the developing zebrafish heart confirms conserved gene expression signatures found in the mammalian SAN and AVN, respectively. Many of the core mammalian SAN/AVN genes are expressed in the developing zebrafish, including *tbx18, hcn4, bmp4, cacna1ab* in the sinoatrial ring [84], and an abundance of genes encoding connexins, T-type Ca^2+^ channel (*cacna1g*), and the pacemaker hyperpolarization channel (*hcn4*) in the AVN [85].

Cardiac physiology in zebrafish aligns closely with mammalian models. The average resting heart rate of humans is 60–90 beats per minute (bpm), while the average heart rate of zebrafish is 120–180 bpm [86]. This characteristic is a considerable advantage over the widely used rodent models like mouse, which have average heart rates of 300–600 bpm. Adult zebrafish basal electrocardiogram (ECG) characteristics are similar to humans, with a distinct P wave, QRS complex, and T-wave [87,88]. As in humans and large animal models, zebrafish have chamber-specific differences in action potential (AP) shape and duration (for examples, please see [87,89,90,91]). In atrial and ventricular cardiomyocytes of zebrafish, the resting membrane potential and AP amplitude are comparable with those observed in humans. Like humans, a clear plateau phase is established in ventricular APs, although a fast phase-1 repolarization is not present. Due to the elevated heart rate in zebrafish, the AP duration in the atrial and ventricular cardiomyocytes is shortened when compared to humans [91].

An estimated 71.4% of human genes have a minimum of one zebrafish ortholog [92]. The fully mapped zebrafish genome includes orthologs of genes encoding for key cardiac ion channels in the human heart [93,94]. Interestingly, some human cardiac ion channels are encoded by non-orthologous genes in zebrafish; for example, the *erg* genes that encode subunits of the functional I_Kr_ channel [95]. Like mammals, zebrafish AP upstroke involves Na^+^ channels, the plateau phase requires L-type Ca^2+^ channels, and repolarization is driven by I_Kr_ channels [91]. Similar fundamental currents are present in zebrafish and human atrial and ventricular cardiomyocytes, yet species-specific differences exist. I_Na_ channel composition and density differs between humans and zebrafish cardiomyocytes [96]. Zebrafish cardiomyocytes lack a transient outward potassium current and a slow delayed rectifier potassium current [91,94,97]. However, using different cell dissociation techniques, both fast and slow components of the delayed K^+^ currents (I_Ks_) have been observed in adult zebrafish ventricular cardiomyocytes [98].

There are distinct morphological differences between zebrafish and mammalian cardiomyocytes. Zebrafish ventricular cardiomyocytes are longer (~100 μm) and narrower (~6 μm) than mammalian cardiomyocytes [91,94,99], more closely resembling neonatal mammals in terms of size. Notably, zebrafish cardiomyocytes lack T-tubules [99] which function in larger mammalian cardiomyocytes to carry extracellular Ca^2+^ deep into the cell. The extensive network of T-tubules facilitates inward Ca^2+^ flow through L-type Ca^2+^ channels and efficient Ca^2+^ release from the sarcoplasmic reticulum. One explanation for this difference could be the smaller size of zebrafish cardiomyocytes; as the cell membrane and myofilaments are closer, T-tubules may not be required for Ca^2+^ handling. Significant differences in zebrafish Ca^2+^ handling have been reported, including a smaller contribution of the sarcoplasmic reticulum to the Ca^2+^ transient, which may in part result from lower expression levels of ryanodine receptors and reduced sensitivity to cytosolic Ca^2+^ concentrations [100]. In contrast to mammals, both L-type and T-type Ca^2+^ channels are expressed in both adult zebrafish atrial and ventricular cardiomyocytes [91]. These differences should be acknowledged given the importance of Ca^2+^ handling and spatial organization of Ca^2+^ channels [101] to cardiac arrhythmias.

## 4. Zebrafish Models of Cardiac Arrhythmia

Cardiac arrhythmias have multifactorial aetiologies. Several zebrafish models with cardiac rhythm phenotypes have been reported (Table 1). Notably, these models provide unique insight toward disease initiation and mechanisms of pathogenesis. Here, we review the categories of genes implicated in the development and pathogenesis of cardiac arrhythmias and highlight the novel biology that these models reveal.

### 4.1. Ion Channels

Many inherited cardiac arrhythmias are linked to rare, autosomal dominant variants in genes encoding ion channels or proteins that regulate ion channel function. These are perhaps the best studied and clearly implicated, given the role of ion channel function in shaping the cardiac action potential. The major cardiac channelopathies are long QT syndrome (LQTS), short QT syndrome (SQTS), Brugada syndrome (BrS), and catecholaminergic polymorphic ventricular tachycardia (CPVT).

LQTS is the most common cardiac channelopathy, characterized by an extended QT interval. Loss-of-function mutations in *KCNQ1* and *KCNH2*, and gain-of-function mutations in *SCN5A* account for ~75% of affected individuals with LQTS [125]. The *breakdance* mutant carries a mutation in *kcnh6a* [102]. Homozygous *breakdance* mutants exhibit a 2:1 atrioventricular block, spontaneous early after depolarizations, and increased action potential duration [112,126]. Using a transgenic approach to express the D1275N mutation in *SCN5A* in zebrafish hearts, Huttner et al. showed that this variant identified in families with conduction defects led to bradycardia, sinus pauses, AV block, and reduced survival [120]. This model was also used to model sick sinus syndrome (SSS) [127]. Acquired LQTS can also be suitably modeled in larval zebrafish, for example, through analyzing cardiotoxic mechanisms (reviewed in [128]). Compounds that block hERG channels act similarly on zERG channels in zebrafish, eliciting bradycardia and 2:1 atrioventricular block [112].

Zebrafish models have also been generated that recapitulate phenotypes observed in SQTS. In humans, gain-of-function variants in *KCNQ1*, *KCNQ1,* and *KCNJ2* affect repolarizing potassium channels leading to the shortening of the action potential duration (APD) [129]. Zebrafish *reggae* mutants carry a gain-of-function mutation in the zebrafish *ether-à-go-go-*related gene zERG, and present cardiac arrhythmia during larval stages. QTc intervals are shortened in both heterozygous and homozygous *reggae* adult fish [113].

Individuals with CPVT have normal resting ECGs and structurally normal hearts but develop episodic syncope during strenuous exercise or during acute emotion. A limited number of genes have been associated with CPVT. Those identified are linked to Ca^2+^ handling, and include *CASQ2, RYR2*, and *CALM1-3* [129,130,131]. While zebrafish orthologs of *casq2* [132] and *ryr2b* [133] are expressed in the zebrafish heart, no models have reported cardiac arrhythmias. Expressing human Calmodulin variants linked to dominantly inherited CPVT did not affect cardiac development or morphology in larval zebrafish. However, β-adrenergic stimulation induced tachycardia and shorter diastolic phase in these models, demonstrating conserved cardiac effects between human and zebrafish [134].

Though Ca^2+^ handling and excitation–contraction coupling are somewhat divergent in zebrafish, disrupting these pathways in zebrafish phenocopy mammalian models. Ca^2+^ handling players like the ATP-dependent Ca^2+^ pump SERCA (zebrafish *atp2a1*, *atp2a2a*), NCX1 (*slc8a1a*), or phospholamban (*plna*) are conserved in zebrafish. In humans, the phospholamban (PLN) variant p.R14del is found in patients with arrhythmogenic cardiomyopathy. Using a *plna* p.R14del line developed in zebrafish [119], Kamel et al. showed that embryonic *plna* p.R14del zebrafish display altered intracellular Ca^2+^ dynamics related to reduced SR Ca^2+^ re-uptake [118]. Adult zebrafish phenotypes recapitulate clinical features of patients carrying *PLN* p.R14del variants, including cardiac remodeling. The authors then showed that istaroxime, a small molecule that stimulates SERCA activity, restores Ca^2+^ handling defects in the model [118]. This example nicely highlights how “humanized” zebrafish can be exploited in drug discovery settings, which is very powerful given that drug treatments are lacking for patients carrying *PLN* p.R14del variants.

Zebrafish can be used to assess pathogenicity of putative human variants using different functional assays. Hyperpolarization-activated, cyclic nucleotide-gated cation currents (I_f_ or I_h_), or pacemaker currents, are generated by members of the hyperpolarization-activated cyclic nucleotide-gated (HCN) channel family. Variants in *HCN4* have been linked to inherited sinus bradycardia [135], which may be a contributing driver of congenital forms of SSS [136]. Morpholino knockdown of *hcn4* in zebrafish leads to bradycardia and prolonged sinus pauses during embryonic stages, which can be rescued by injecting wild-type *hcn4* mRNA [110]. This assay was used to test the functional consequence of variants identified in individuals with congenital SSS and enabled pathogenicity classification for several novel variants of unknown significance [110]. *KCNMA1* encodes for the α-subunit of the large-conductance Ca^2+^-activated K^+^ channel, K_Ca_1.1 and has been linked with AF. A knockdown approach on the zebrafish homolog gene, *kcnma1b* resulted in sinus bradycardia with dilatation and reduced contraction of the atrium and ventricle [114].

Additional conduction mutants identified from large-scale screening efforts have been mapped to genes affecting cardiac electrophysiology. The *slow mo* (*slwm*) mutant exhibits bradycardia from the onset of cardiac contraction [137]. While a slow component of I_f_ remains unchanged in *slwm* mutant cardiomyocytes, the fast kinetic amplitude is greatly reduced. Bradycardia is also observed in the adult *slwm* mutants but without altered cardiac morphology [138]. The *hiphop* (*hip*) mutant carries a missense mutation in the Na^+^/K^+^-ATPase α1-subunit (*atp1a1a.1*) and has irregular and reduced heart rate [102,103]. The *hip* mutation acts as a hypomorphic allele leading to prolonged QT interval and refractoriness, indicating that Na^+^/K^+^-ATPase is essential for heart rate regulation by prolonging myocardial repolarization. The zebrafish mutant *island beat* (*isl*) [15] was mapped to the voltage-dependent L-type Ca^2+^ channel α1C subunit (*cacna1c*) [104]. This mutant exhibits uncoordinated contraction of the atrial cardiomyocytes while the ventricle appears silent. In another forward genetic screen, *grime* mutants were identified with bradycardia, skipped ventricular beats, and irregular heartbeats without defects in cardiac morphology [123]. This phenotype is caused by a mutation in *tmem161b,* which encodes a transmembrane domain protein localizing to excitatory cell membranes. Functionally, Tmem161b is required for correct action potential polarization and regulates I_Kr_ and I_CaL_ currents in cardiomyocytes.

### 4.2. Developmental Transcription Factors

AF is the most prevalent cardiac arrhythmia and increases markedly with age. AF is highly heritable and linked to both common genetic variants in the general population (i.e., risk alleles) and rare, highly penetrant mutations in familial forms of the disease. From GWAS and exome sequencing studies, transcription factors have emerged as important drivers of disease pathogenesis, including *TBX5*, *GATA4*, *NKX2*-5, *PITX2*, and *ISL1* [7,11,139,140,141]. Notably, many of these genes are required during cardiac development (Figure 2).

The SAN transcription factors Shox2 and Is1l are crucial for the development and maintenance of pacemaker cells in the zebrafish heart [142]. In zebrafish, mutations in *isl1* result in sinus arrhythmia development due to loss of pacemaker cells; bradycardia is observed due to prolonged pauses between heartbeats with an irregular rhythm overall [67,111]. Mutations in the homeobox gene *SHOX2* have been associated with early-onset AF [143,144]. Zebrafish with cardiac-specific overexpression of the putative human variants were successfully used to demonstrate the pathological potential of a subset of these novel variants [145]. Similar to morpholino knockdown of *shox2* [144], expression of the human variants leads to pericardial edema and reduced heart rate.

Transcription factors involved in early conduction patterning have been studied in zebrafish. Nkx genes function to maintain ventricular identity [26,146]. *nkx2.5* mutant embryos have elevated heart rate and decreased heart rate variability compared to wild types, suggesting that Nkx2.5 establishes normal heart rate variation parameters in zebrafish [116]. T-box transcription factor family member Tbx5 is key for proper development of the heart, eye, and pectoral fin buds [147,148]. A lethal recessive mutation in *heartstrings* (*hst*) appears to completely terminate *tbx5a* function, resulting in a lack of pectoral fin development and cardiac dysfunction [122]. *hst* hearts exhibit severe bradycardia from the onset of contraction and fail to complete the process of heart looping. The heart becomes stretched and thin by 48 hpf, and contraction arrests between 72 and 96 hpf [122]. In addition to AF, TBX5 is also implicated in Holt–Oram syndrome, a condition characterized by congenital defects in the upper limbs and the heart, the latter of which often includes cardiac conduction disease and septation defects [149,150].

As identified in several GWAS, variants at the 4q25 locus confer an increased risk for AF [151]. These variants are in an intergenic desert that influences the expression of the nearby gene, *PITX2* [152,153]. Loss of *pitx2c* in zebrafish leads to adult cardiac phenotypes reminiscent of pathologies observed in AF patients, including arrhythmia, atrial conduction defects, sarcomere disassembly, and dysregulated cardiac energetics [117]. In larval zebrafish, sarcomere and metabolic defects are observed prior to the onset of cardiac arrhythmia, and treatment with the antioxidant *N*-acetyl cysteine reduced the severity of cardiac arrhythmia in larval hearts. These data point to an early cardiomyopathy phenotype that may be exacerbated by reactive oxygen species (ROS) levels. As increased oxidative stress is associated with AF in mouse and human [154,155], it may suggest that these factors are important modifiers rather than co-morbidities of disease.

The *hobgoblin* (*hob*) zebrafish mutant was found in a genetic screen using a genetically-encoded Ca^2+^ biosensor (GCaMP) zebrafish reporter line [64]. *hob* mutants display an AV block at 48 hpf followed by a silent ventricle at 96 hpf [64]. The *hob* mutation was mapped to the gene encoding Tcf2, a transcription factor expressed in the AVC and outflow tract of the developing heart. The *slipjig* (*sli*) mutant was found in the same screen, exhibiting continuous peristaltic pumping instead of sequential beating of the atrial and ventricular chambers. *sli* mutants carry a mutation in the gene encoding the transcription factor Foxn4, which is expressed in the AV myocardium [108]. Molecular analysis showed dysregulated expression of AVC markers including *tbx2b*, *bmp4*, and *versican* in *sli* embryos, suggesting that these are important transcriptional targets of Foxn4 in the heart. Additional zebrafish mutants with cardiac conduction phenotypes such as *mobitz*, *elektra*, *daredevil*, *bullseye*, and *kingpin*, found during the same genetic screen [64], were not mapped to specific genes.

### 4.3. Cardiac Muscle

Cardiac structural genes have been associated with arrhythmias like early-onset AF and arrhythmogenic cardiomyopathy, including genes encoding the sarcomere proteins myosin light chain 4 (*MYL4*) [10,105], titin (*TTN*) [124], cytoskeletal heart-enriched actin-associated protein CHAP (*SYNPO2L*) [156], and desmosomal proteins [7,157]. Perturbing the function of these genes in zebrafish leads to atrial cardiomyopathy phenotypes that precede the onset of arrhythmia, highlighting a potential mechanism of arrhythmogenesis.

Missense variants in *MYL4* have been identified in familial early-onset AF cases. A zebrafish model of one identified variant, *MYL4* p.Gly11Lys (E11K), tested the pathogenicity of the corresponding mutation E17K in the zebrafish orthologue *cmlc1* [105]. E17K adult transgenics exhibited severe bradycardia and slowed atrial conduction with arrhythmia, as well as structural alteration including enlarged atria. Sarcomere disruption was observed at larval stages, evidenced by disorganized myofibrils, abnormal H-zones, and mostly absent Z-discs, while heart rate and cardiac output were unaffected at these stages [105]. Another study, *myl4* loss-of-function mutant larvae exhibited prolonged action potential duration, altered Ca^2+^ handling, and abnormal localization of Cx43 [106]. The latter finding was also confirmed in atrial biopsies from patients who developed AF following surgery or those with permanent AF. This study also established a significant genetic interaction between *MYL4* mutant alleles and common risk alleles at the *PITX2* locus [106], which is very interesting given the similar observations in *pitx2c* mutant larvae that display disorganized sarcomeres prior to the onset of cardiac arrhythmia [117].

AF GWAS data identifying *SYNPO2L* also points to a role for structural proteins predisposing the heart to arrhythmogenesis. Morpholino knockdown of *chap1* and *chap2,* the zebrafish orthologues of *SYNPO2L*, resulted in aberrant heart development, reduced cardiac contractility, and sarcomere defects [158]. No studies on the electrical activity in zebrafish have been reported, but conduction defects were observed in a *CHAPb* transgenic mouse model [159]. Defective sarcomere assembly during early development is also evident in a zebrafish mutant carrying an N-terminal truncated titin variant [124,160]. Homozygous mutant *ttn.2* larval hearts lack Z-discs. Heterozygous *ttn.2* adults present highly disorganized sarcomeres with shortened myofibrils and absent I-bands and M-lines, as well as increased atrial fibrosis and electrophysiological defects. Together, these observations further strengthen the emerging hypothesis that atrial cardiomyopathy predisposes for cardiac arrhythmias like AF.

The desmosome is a specialized cell junction complex that mechanically integrate desmosomal cadherins with the cytoskeleton. Mutations in desmosomal proteins including desmoglein-2, desmocollin-2, plakophilin-2 are linked to arrhythmogenic cardiomyopathy [157]. No genetic mutants have been reported for *pkp2* in zebrafish, but morpholino knockdown of *pkp2* reduces heart rate and causes additional cardiac development phenotypes [161]. Knockdown of zebrafish orthologs *dspa* and *dspb* reduces desmosomal junctions, similar to what is reported in ARVC patients [162]. Connexins, or gap junction proteins, are also necessary for correct conduction system patterning in zebrafish. The *dococ* (*dco)* mutant, which carries a mutation in Connexin 46 (*gja3/cx46*), exhibits asynchronous ventricular contraction and AV conduction block [109]. Connexin 43 (*cx43*) deficient embryos have reduced heart rate, arrhythmia, and develop heart failure [107].

### 4.4. Metabolic Regulators

Cardiac mitochondria control the metabolic processes required for cardiac function, producing over 95% of the energy in the heart. Oxidative phosphorylation is finely tuned to adapt to the changing metabolic demand of cardiomyocytes. Given the importance of mitochondria for cardiac energetics, it follows that metabolic alterations are reported in cases of cardiac dysfunction, including cardiac arrhythmias like AF and ventricular arrhythmias. However, it is challenging to distinguish between primary drivers of disease and secondary co-morbidities.

Oxidative stress has been implicated as a potential arrhythmogenic mechanism in AF, which has been further explored in zebrafish models. It was reported that AF patients displayed marked upregulation of NADPH oxidase isoform 4 (*NOX4*) [163]. Interestingly, an embryonic zebrafish *NOX4* overexpression model showed significant heart rate variability, which could be attenuated by co-injection with a *NOX4* morpholino or with treatment with ROS scavengers, including superoxide dismutase. These data suggest that NOX4 activation and consequent NADPH-driven ROS production is a novel mechanism underlying the development of cardiac arrhythmia [163]. Increased oxidative stress has also been suggested as a potential factor in Brugada syndrome (BrS), which is typically considered a channelopathy. Between 20 and 25% of BrS patients carry variants in *SCN5A,* leading to a loss of function in the Nav1.5 sodium channel [164]. Additional BrS-related genes encode the β-subunits of Nav1.5 [165,166]. Recently, a genome-wide CNV study in BrS patients without *SCN5A* variants identified *GSTM3* as a novel genetic modifier in BrS [167]. *GSTM3* encodes a glutathione S-transferase that protects cells from oxidative stress. Adult male *GSTM3* heterozygous and homozygous mutant zebrafish have ventricular arrhythmia and pharmacological responses to flecainide and quinidine comparable to BrS ECG parameters [167], providing further evidence that increased oxidative stress can lead to cardiac arrhythmia. In zebrafish, loss of the mitochondrial Ca^2+^ uniporter MCU leads to changes in adult atrial morphology, including sarcomere disassembly, as well as conduction defects [115]. Loss of MCU attenuates mitochondrial Ca^2+^ uptake, leading to high levels of ROS, which could be indicative of pathological remodelling.

The question of whether oxidative stress is an arrhythmogenic mechanism that may be modulated pharmacologically has also been explored in zebrafish. In our studies of *pitx2c* mutant larvae, transcriptomics data indicated aberrant gene expression in oxidative phosphorylation pathways prior to when cardiac arrhythmia was observed [117]. In line with this, *pitx2c* mutants had increased ROS levels, and antioxidant treatment could diminish the arrhythmic burden. The efficacy of mitochondrial Ca^2+^ uptake enhancers to restore cardiac rhythm was also recently reported [168]. In a screen using the *tremblor* mutant, a model for Ca^2+^ induced cardiac arrhythmia [121], ezetimibe and disulfiram, drugs that stimulate SR-mitochondria Ca^2+^ transfer, were shown to rescue cardiac arrhythmia [168]. Most common anti-arrhythmic compounds target ion channels, consequently leading to proarrhythmic side effects. Exploring molecules that act on other arrhythmogenic substrates could lead to developing promising new candidates to explore.

## 5. Techniques for Assessing Cardiac Rhythm and Function in Embryonic and Adult Zebrafish

### 5.1. Tools to Study Cardiac Rhythm at Embryonic Stages

Due to its amenability to live imaging and genetic manipulation, the zebrafish model provides a great opportunity for understanding the genetic and molecular mechanisms underlying cardiac arrhythmia. Here we summarize the different techniques used to assess, image, and control the rhythmicity of heart contractions in zebrafish embryos and adults.

The zebrafish embryo is easy to image due to its transparency, and light microscopy is suitable to detect early arrhythmia. High-speed acquisition of the beating heart is key to the identification of arrhythmia. One of these techniques, spinning disk microscopy, provides many attractive advantages for imaging heart contractions in vivo during development due to its speed with higher frame rates. The high speed allows the imaging of multiple samples in a short amount of time, making it an excellent instrument for high-throughput chemical screening assay in zebrafish embryos [169]. After acquiring heartbeat movies, a kymograph, a plot representing spatial position over time, can be generated to quantify heart rate, heart rate variability, and cardiac output in larvae [67,117,123,170]. Automation of this process using algorithms that have been developed to quantify heart rate variability like ZebraBeat [171], MUSCLEMOTION [172], and ImageJ/FIJI-based tools [86,173] to analyze video data can improve operator outcomes in terms of speed.

Light-sheet microscopy is another fluorescence microscopy technique suitable to detect arrhythmia. It uses a plane of light to optically section and views tissues at a cellular resolution. Light-sheet microscopy presents the advantage of deep imaging with a thin plane of light, limiting phototoxicity and photobleaching [174]. By combining this approach with optogenetics, a technique in which channels can be controlled with light, Arrenberg et al. created an optically controlled pacemaker by expressing halorhodopsin and channelrhodopsin in zebrafish cardiomyocytes [66]. Using these tools, the cardiac pacemaker was mapped using a patterned illumination to localize the areas sensitive to hyperpolarization at the inflow tract and AV canal during early development. By varying the light intensity, the authors manipulated cardiac rhythm to simulate tachycardia, bradycardia, atrioventricular blocks, and cardiac arrest [66].

One obvious challenge while imaging cardiac rhythm is the constant motion of the heart. Different solutions are possible to overcome the limitation for long time-lapse in larvae, such as preventing heartbeat by injecting a morpholino targeting the cardiac troponin T gene *tnnt2a* [175]. The heart may be transiently stopped for imaging using tricaine, an anesthetic drug that prevents sodium ions from entering the cell and thus eliminates the action potential, or blebbistatin, which uncouples excitation–contraction by inhibiting myosin ATPase activity. However, these approaches are limited due to their effect on heart development and physiology. One exciting possibility is the development of algorithms that synchronize and acquire the entire beating heart to perform 3D reconstruction devoid of motion artifacts [176].

By taking advantage of the permeability of the zebrafish embryo, chemical dyes can be used to image heartbeats by indicating electrical potential changes using di-8-ANEPPS or Ca^2+^ dynamics such as Fura-2 [65]. They present the advantages of being relatively easy to use, although cell specificity and poor suitability for chronic imaging are drawbacks. Biosensors, or genetically encoded reporters, have been developed for use in zebrafish due to their reduced toxicity and ability to target specific cell types. Such biosensors can be used to visualize signaling pathways essential for cardiac rhythm, including Ca^2+^ dynamics using GCaMPs, a synthetic fusion of green fluorescent protein (GFP) and calmodulin as the sensing element [64,177]. Recently, fluorescence resonance energy transfer (FRET)-based Ca^2+^ indicators based on troponin C variants, Twitch proteins, were tested transiently in zebrafish and showed promising results as new genetically encoded Ca^2+^ indicators [178]. These new biosensors have been described as having potentially less interference with Ca^2+^ regulatory elements than the calmodulin-based biosensors. It would be interesting to generate stable transgenic lines from these genetic constructs to test them for cardiac arrhythmias. Another recently developed direction used bioluminescence as an approach to detect arrhythmia by fusing GFP with aequorin [179]. This tool is especially well suited to prolonged imaging without artificially stopping the heart.

Genetically encoded voltage reporters have been developed and tested in zebrafish. One of the first zebrafish applications used a FRET strategy called Mermaid [180,181]. Mermaid is derived from a tunicate voltage-sensitive phosphatase in which the change in voltage triggers conformational changes in the voltage-sensing domain. The Mermaid construct was able to report the voltage dynamics of a beating heart even though it could not define the action potential waveform. Moreover, its FRET spectra excluded its use in combination with a GCaMP sensor. To overcome this limitation, a mutated Archaerhodopsin protein with a far-red spectrum was used as a voltage indicator in combination with a GCaMP sensor [182]. This construct, named CaViar, allows for simultaneous mapping of membrane voltage and Ca^2+^ dynamics in the heart and the measurement of the cardiac action potential. Recently, a voltage-sensitive fluorescent protein sandwiched between a FRET pair of proteins (VSFP-butterfly) was used to characterize cardiac arrhythmia phenotypes in the *tmem161b* mutant [123]. In addition to imaging-based approaches, cardiac ion channel behavior may be recorded from larval hearts [85,137,183] or dissociated adult cardiomyocytes [123,184] using patch-clamp techniques.

### 5.2. Applications for Adult Cardiac Rhythm Phenotyping

Cardiac rhythm can be monitored in adult zebrafish even though it presents more challenges due to the opacity of the animal. Echocardiography is an ultrasound imaging method that uses a high-frequency transducer directly applied to the body of the anesthetized zebrafish. This technique is non-invasive and enables quantification of cardiac output parameters including chamber area, fractional area change, and fractional shortening using brightness mode (B-mode) [117]. Pulsed-wave Doppler imaging provides information on blood flow and valve function [53,185]. ECG is the most common method to evaluate cardiac electrophysiology clinically and may be applied in zebrafish. Electrodes are placed on top of the cardiac region to record the T wave, P wave, and QRS complex. The measurement of the P wave duration and PR intervals, representing atrial depolarization, can indicate atrial conduction defects, as has been reported for several models [87,117,124]. Recently, simultaneous bipolar dual-lead ECG recordings that more closely mimic the clinical situation revealed three electrical heart axes, which will be relevant to avoid misinterpretation of the clinical relevance of the adult zebrafish for future arrhythmia studies [90]. Magnetic resonance imaging (MRI) uses a magnetic field and radio waves to provide detailed images of the organs and tissues of the body. MRI has been increasingly employed in non-aquatic animals for cardiovascular disease, and its use has recently been shown to be possible in anesthetized zebrafish [185,186]. MRI presents the advantage of being highly resolutive, non-invasive, and suitable for morphological characterization of the cardiac chambers in zebrafish. The different methods described here provide a wide range of possibilities to measure cardiac rhythm in zebrafish embryos and adults and evaluate cardiovascular performance, gene function assays, and high content drug screening.

## 6. Outlook

The combination of genetic studies and functional genomics in zebrafish has broadened our knowledge on mechanisms of cardiac disease. Genetic studies in patients with cardiac arrhythmias have boomed, yet there exists a paucity of functional modeling of newly uncovered variants. As the ability to knock-in genetic sequences becomes more feasible and higher throughput in zebrafish [119,187,188,189,190], models expressing human variants will provide valuable insight into disease mechanisms. This approach is an especially powerful to elucidate the pathogenicity of specific variants, as highlighted above. One major challenge will be assessing the deleterious effects of non-coding variants. Often, these regions show less conservation amongst species, and thus are more challenging to interpret. However, functional biological evidence may be garnered by mutating putative genes in zebrafish identified from synteny, eQTL analysis, or chromosome capture techniques. Furthermore, understanding how epigenetic alterations, including methylation, histone modification, and chromatin remodeling, contributes to disease risk [11] will be an exciting avenue to explore.

As genetic variation may predict response to different therapies, zebrafish may be used in suppressor screens to identify novel chemical modulators of disease phenotypes or new arrhythmogenic substrates. Zebrafish carrying *myl4* or *plakoglobin* mutations have been used in small molecule screens to identify modifiers of cardiac arrhythmia, and have identified conserved disease mechanisms in both zebrafish and mammalian models [106,126,183,191]. It will be exciting to see how zebrafish mutants carrying putative human variants respond in similar screens. These findings may provide an avenue to pharmacogenetically guide therapy for cardiac rhythm disease to yield more targeted and effective treatment strategies.

While the suitability of zebrafish to study arrhythmia is evident, caveats and limitations as mentioned above should guide interpretation as with all models of human disease. Even though zebrafish possess a smaller two-chambered heart, we have learned many lessons that have been extrapolated to understanding human heart development, disease, and regeneration. However, important species-specific parameters, including ionic currents or dependence on sarcoplasmic reticulum Ca^2+^ stores in excitation–contraction coupling, should be acknowledged when modeling cardiac arrhythmias and translating these findings to human disease. Nonetheless, as more genomic sequencing data from patients becomes available, the zebrafish is poised to make impactful observations to impact human health and personalized medicine.

## Figures and Tables

**Table 1:** Zebrafish models of cardiac arrhythmia.

**Model/** **Gene**	**Allele**	**Cardiac Defect**	**Clinical** **Arrhythmia**	**Human Ortholog**	**Ref.**
*atp1a1a.1*	*hiphop (tx218)*	3:1 ratio of atrial contraction to ventricular contraction, bradycardia, and AV-block.	LQTS	*ATP1A1*	[102,103]
*cacna1c*	*island beat (m379,* *m458*, *m231)*	Silent ventricle, uncoordinated contraction of the atrium.	AF	*CACNA1C*	[15,104]
*cmlc1* *myl4*	*s977* *bw24*	Bradycardia, slow conduction in enlarged atrium, sarcomere disorganization.	AF	*MYL4*	[105,106]
*cx43 (gja1b)*	Morpholino	Bradycardia, AV-block, and fibrillation.	AF	*GJA1*	[107]
*foxn4*	*slipjig s644)*	Peristaltic contraction with no AV delay.		*FOXN4*	[64,108]
*gja3/cx46*	*dococ (s215, s226)*	Uncoordinated conduction and contraction within the ventricle.		*CX46*	[109]
*hcn4*	Morpholino	Bradycardia and prolonged cardiac pauses.	SSS	*HCN4*	[110]
*isl1 (K88X mutant)*	*sa0029*	2 dpf: bradycardia due to impaired SA node function. 3–4 dpf: sinus block.	SSS	*ISL1*	[67,111]
*kcnh6a (zerg)*	*breakdance (tb218)*	2:1 ratio of atrial to ventricular contraction, bradycardia, reduced cardiac output, and AV-block due to impairment of I_Kr_ channel.	LQTS	*KCNH6 (hERG)*	[102,112]
*kcnh6a (zerg)*	*reggae*	Intermittent atrial fibrillation and acceleration of cardiomyocyte repolarization.	SQTS	*KCNH6 (hERG)*	[113]
*kcnma1b*	Morpholino	Decreased contraction of heart chambers, sinus bradycardia.	AF	*KCNMA1*	[114]
*mcu*	*la2446*	Cardiomyopathy. Thin, dilated atrium, small ventricle with restricted blood flow, swollen mitochondria. Heart rate variability.	SSS	*MCU*	[115]
*nkx2.5*	*vu176, vu413*	Reduced heart rate variation, increased heart rate.	CHD	*NKX2-5*	[116]
*pitx2c*	*ups6*	Embryonic: arrhythmia, sarcomere disorganization, increased ROS. Adult: extended P-wave and PR-interval, fibrosis, sarcomere disorganization.	AF	*PITX2*	[117]
*pln*	*hu10742*	Adult: structural remodeling, immune cell infiltration, contractile defects, AP alternans, altered Ca^2+^ handling	ACM	*PLN*	[118,119]
*scn5a*	human variant	Bradycardia, sinus pauses, AV-block.	LQTS	*SCN5A*	[120]
*slc8a1a* *(ncx1)*	*tremblor (tc318d, te381b, m116, m139, m158, m276, m736)*	Fibrillation from onset of contraction (more prominent in the atrium than the ventricle). Absent circulation.		*SLC8A1* *(NCX1)*	[15,102,121]
*tbx5a*	*heartstrings (m21)*	Slight bradycardia evident during initial heart tube stage. Heart fails to loop, contractility declines, and pericardial edema develops.	Holt–Oram syndrome	*TBX5*	[122]
*tcf2*	*hobgoblin (s634)*	AV block at 48 hpf, silent ventricle at 96 hpf.		*TCF2*	[64]
*tmem161b*	*grime (uq4ks)*	Bradycardia, skipped ventricular beats, increased heart rate variability	LQTS	*TMEM161B*	[123]
*ttn.2*	*sfc9*	Atrial fibrosis, compromised sarcomere assembly in atrium and ventricle, lengthened PR interval.	AF	*TTN*	[124]
	*mobitz (s466)*	AV block, sinus pause at 120 hpf.			[64]
	*elektra* (*s587)*	AV block.			[64]
	*daredevil (s275, s563)*	AV block, silent ventricle at 120 hpf.			[64]
	*bullseye (s885)*	No heartbeat at 24 hpf, AV block at 36–48 hpf.			[64]
	*kingpin (s886)*	Atrial and ventricular fibrillation			[64]

**Model/**

**Gene**

**Allele**

**Cardiac Defect**

**Clinical**

**Arrhythmia**

**Human Ortholog**

**Ref.**
